# Synaptic Loss in Multiple Sclerosis: A Systematic Review of Human Post-mortem Studies

**DOI:** 10.3389/fneur.2021.782599

**Published:** 2021-11-29

**Authors:** E. E. Amelie Möck, Eveliina Honkonen, Laura Airas

**Affiliations:** ^1^Division of Clinical Neurosciences, Turku University Hospital and University of Turku, Turku, Finland; ^2^Turku PET Centre, Turku University Hospital and University of Turku, Turku, Finland

**Keywords:** synapses, synaptic density, synaptic loss, gray matter, multiple sclerosis, review, smoldering

## Abstract

**Background:** Gray matter pathology plays a central role in the progression of multiple sclerosis (MS). The occurrence of synaptic loss appears to be important but, to date, still poorly investigated aspect of MS pathology. In this systematic review, we drew from the recent knowledge about synaptic loss in human post-mortem studies.

**Methods:** We conducted a systematic search with PubMed to identify relevant publications. Publications available from15 June 2021 were taken into account. We selected human post-mortem studies that quantitatively assessed the synapse number in MS tissue.

**Results:** We identified 14 relevant publications out of which 9 reported synaptic loss in at least one investigated subregion. The most commonly used synaptic marker was synaptophysin; non-etheless, we found substantial differences in the methodology and the selection of reference tissue. Investigated regions included the cortex, the hippocampus, the cerebellum, the thalamus, and the spinal cord.

**Conclusion:** Synaptic loss seems to take place throughout the entire central nervous system. However, the results are inconsistent, probably due to differences in the methodology. Moreover, synaptic loss appears to be a dynamic process, and thus the nature of this pathology might be captured using *in vivo* synaptic density measurements.

## Introduction

Multiple sclerosis (MS) is a widespread chronic disease of the central nervous system (CNS), which affects the white and gray matter (GM) of the brain and the spinal cord (1, 2). To date, the complex interplay of underlying pathological processes is still insufficiently understood. Besides acute inflammatory events, chronic smoldering processes appear to drive the disease progression relapse independently (3). Although traditionally white matter alterations have been dominating as the core feature of MS, considering also other measures, such as atrophy and GM alterations, has shown to be highly valuable in predicting long-term disability and cognitive impairment (4–9). Histopathological examinations of the GM report neuroaxonal loss, transected dendrites, and synaptic loss (10–14). Notably, GM alterations appear at all disease stages; however, GM lesions are predominantly present in progressive disease phases (15, 16).

To date, insights into the synaptic density of the human CNS of patients with MS are limited to a relatively small number of post-mortem studies. Synaptic loss appears in different areas of the cortex, such as the frontal gyrus, precentral- and postcentral gyrus (13) the frontotemporal cortex and insular cortex (17). Evidence for a reduction has been found also in the hippocampus (11, 12, 18), the thalamus (19), the cerebellum (20), and in the spinal cord (21). A change of in synaptic density has been identified primarily in lesional GM, but in a growing number of studies also in normal appearing gray matter (NAGM), going beyond areas of demyelination and exceeding neuronal loss (17, 20, 21). Thus, it can be hypothesized that the processes driving synaptic loss are surpassing secondary degeneration and do not solely follow neuronal death and demyelination.

Clearly, synaptic pathology includes not only synaptic loss but also more subtle functional disturbances, as well as alterations of electrical synapses. Considering the important physiological role of chemical synapses in neurotransmission and network dynamics, synaptic loss might contribute to functional impairment of MS patients. The aim of this review is to systematically assess the research reporting on quantitative changes of chemical synapses in distinct sites of the human CNS. A comprehensive analysis of available data will be valuable for the interpretation of regional measures in a broader context and to provide a reference for further research. A better understanding of GM pathology in MS, with synaptic loss accounting for a central component, is urgently needed considering the great impact on physical disability and cognition and moreover, the lack of effective treatment, especially for progressive stages of the disease (22).

### Synaptic Markers

Most markers used for the quantification of synapses in MS brains represented exclusively the presynaptic site:

Synaptophysin, synapsin-1, and synaptic vesicle 2 (SV2) are specific proteins for synaptic vesicles in the brain (23). Synaptophysin, a 38-kDa glycoprotein (24), is the most abundantly targeted synaptic protein in human MS research and is also commonly used for synaptic quantification in the context of other diseases (25). Like other presynaptic vesicle proteins, it represents neuronal afferent connectivity and has shown to be a reliable marker for synaptic quantification (26). Nonetheless, sensitivity and outcome can vary significantly depending on the applied technique to measure synaptophysin-immunoreactivity (27). Synapsin-1 is a presynaptic synaptic vesicle phosphoprotein belonging to the synapsin family, which comprises five isoforms (28). Synapsins are present in large numbers in synaptic vesicles and are thought to play an important role in regulating neurotransmitter release (29). In the synaptic vesicle 2 (SV2) family, three isoforms are included, out of which only SV2A is ubiquitously expressed throughout the adult brain (30). Likewise located in synaptic vesicles is the protein synaptotagmin. It is expressed in 17 isoforms and involved in the regulation of membrane-trafficking (31, 32). Another presynaptic structure is the vesicular glutamate transporter 2 (VGluT2). It is mainly localized in the thalamus and the brain stem (33) and specific to retinogeniculate presynaptic input in the lateral geniculate nucleus of the thalamus (19).

Other targeted synaptic molecules are associated with both, pre- and postsynaptic structures, to the synaptic gap or the postsynaptic site:

Calcium/calmodulin-dependent serine protein kinase (CASK) is a membrane-associated protein (34) with pre- and postsynaptic occurrence (35). Neurexin-1 and Neuroligin-1 are part of the specialized synaptic adhesive junction (36). While neurexin-1 is localized at the presynaptic site, neuroligin-1 is considered to be a postsynaptic protein (37). The postsynaptic density protein 95 (PSD-95) belongs to the postsynaptic density fraction (38) and is associated with synapse stability and development (39). The mammalian microtubule-associated protein 2 (MAP2) is enriched in dendrites and is thought to be important for neuronal plasticity (40, 41).

## Methods

### Search Results

The systematic literature search yielded 236 publications, out of which we identified 11 as relevant. Additionally, we included three papers from manual search in the literature references of relevant publications. There was insufficient data to perform a meta-analysis since the minimum amount of five independent datasets required in order to reliably achieve powers from random-effects meta-analyses (42) was not given for any CNS region.

### Search Strategy, Study Selection, and Data Extraction

**Figure 1:**
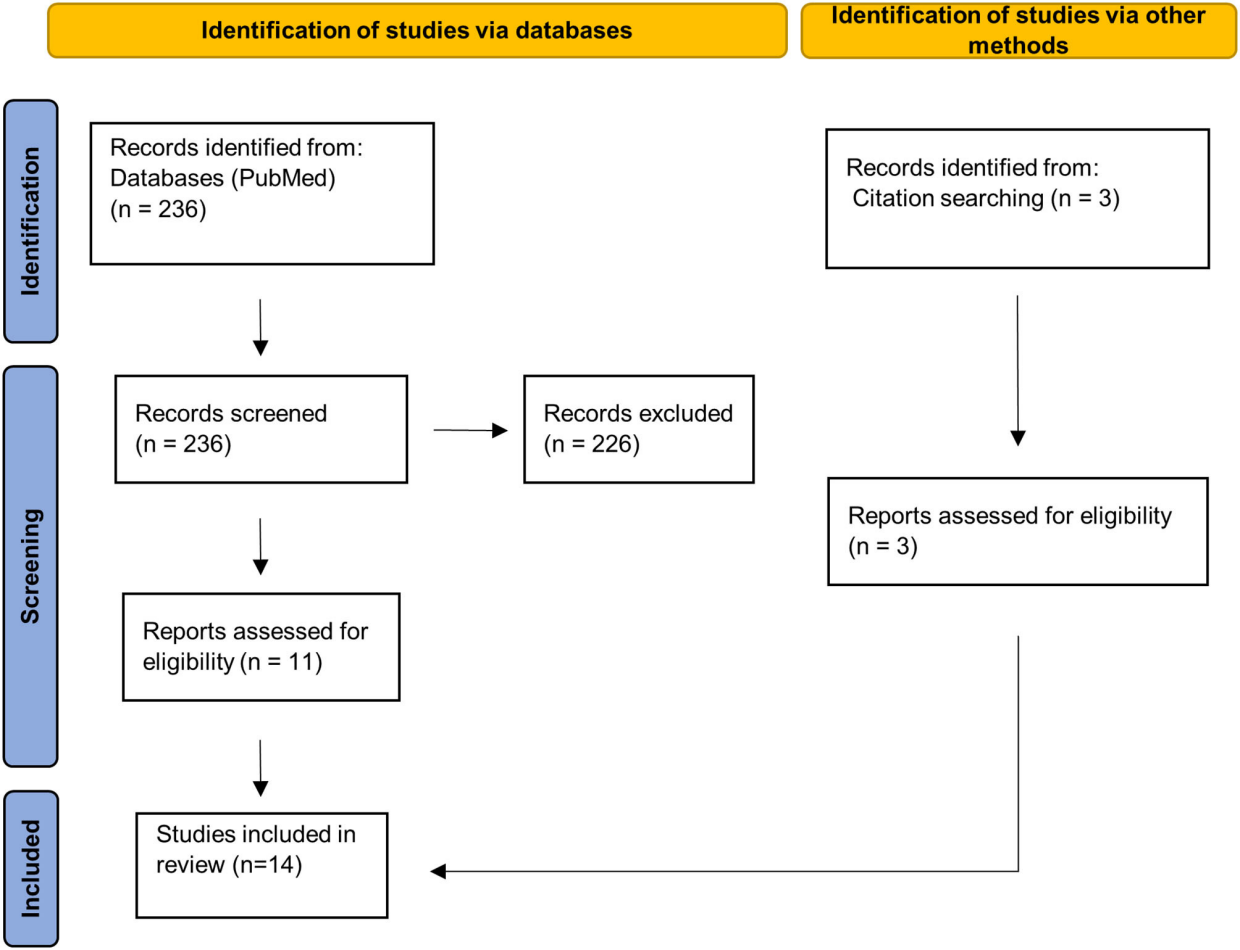
PRISMA 2020 flow diagram. Page et al. (43). For more information, visit: http://www.prisma-statement.org/.

This systematic review follows the PRISMA guidelines. All publications available from 15 June, 2021 were taken into account. The search was conducted with PubMed (Figure 1). Each of the following listed search terms was individually combined with “multiple sclerosis.” The respective search term and MS were connected using “OR” with the subsequent search term and MS (e.g., synaptic density MS OR dendrite spine loss MS OR, etc.). List of search terms: synaptic density, dendrite spine loss, synaptic loss, synaptophysin, synaptotagmin, syntaxin, synaptobrevin, synapsin, PSD-95, and MAP2. The reference sections of retrieved articles were additionally searched for further relevant publications. Relevant publications were identified based on the title and the abstract. Two researchers independently screened potential publications. Discrepancies in the selection of studies were resolved by discussion.

Criteria for inclusion: (1) human post-mortem studies, assessing synapses quantitatively in patients with MS directly in the CNS, (2) available comparison of the results either between MS lesional and NAGM or MS GM and HC, and (3) publication in English.

Criteria for exclusion: (1) studies exclusively in animal models for MS, (2) studies assessing synaptic alterations without directly addressing the question of quantitative changes of synapses, and (3) review articles.

Extracted data included the area of the CNS, method of quantification, method of myelin staining, sample size of MS population and HC population, and relevant associated information such as gender, mean age, mean disease duration, characteristics of MS disease (e.g., RRMS, SPMS, etc.), EDSS, post-mortem interval, matching for age, and sex. Data only available in the form of a plot were extracted using the software WebPlotDigitizer, Version 4.4 (https://automeris.io/WebPlotDigitizer/).

## Results

### Cortex

Six studies examined the synaptic density in different cortical areas, three of which described significant synaptic loss (13, 17, 44), whereas the others found no overall reduction (45–47) (Table 1).

**Table 1:** An overview of publications that investigated the synaptic density in the cortex along with extracted data.

**Publication**	**CNS area**	**Synaptic staining**	**Investigated anatomical subregion**	**Myelin detection**	**Result**	Population^*^	**Specifics MS**	**Mean-age (years)**	**Mean disease duration (years)**	**Disability** **(EDSS)**	**Post-mortem interval (h)**
Vercellino et al. (46)	**Cortex** - whole cortical section	IHC - SYN (OD)	Synaptic vesicle (presynapse)	MBP	No change (M vs. DM)	MS: 6 (5 female) HC: 0	3 RRMS, 3 SPMS	MS: 52 (39–66)	14.3 (8–29)	7.2 (6.0–9.0) (including 5 patients, 1 NA)	NA
Wegner et al. (13)	**Cortex** - superior frontal gyrus - precentral gyrus - postcentral gyrus	IHC - SYN (immunoautoradiography) - GAP-43 (growth marker)	Synaptic vesicle (presynapse)	MBP	Reduction in leucocortical lesions	MS: 9 (7 female) HC: 9, age- and sex- matched	NA	MS: 56 (32–69) HC: 59.5 (25–72)	NA	NA	MS: 50 (16–96) HC: 50.5 (16–96)
Vercellino et al. (47)	**Cortex** - prefrontal cortex - temporal cortex - insular cortex - cingulate cortex	IHC - SYN (OD)	Synaptic vesicle (presynapse)	MBP	Overall no change (M vs. DM), focal loss in colocation with activated microglia	MS: 10 (9 female) HC: 0	3 RRMS, 7 SPMS	MS: 53.4 (27–66)	17.44 (8–30)	NA	MS: < 36
Jürgens et al. (17)	**Cortex** - frontotemporal cortex - insular cortex - occipital cortex	Golgi-Cox impregnation (confocal microscopy)	Dendrite spine	MBP	Reduction in M and DM	MS: 8 (5 female) HC: 8 (3 female), age-matched	8 SPMS and PPMS	MS: 56.75 ± 10.48 HC: 69.88 ± 11.33	>10	NA	MS: NA HC. NA
van Olst et al. (45)	**Cortex** - insular cortex - cingulate cortex	IHC - SYN (confocal microscopy)	Synaptic vesicle (presynapse)	MOG	Overall no change (vs. control), focal loss in collocation with activated microglia	MS: 20 (11 female) HC: 6 (1 female)	20 SPMS	MS: 48.9 (35–65) HC: 59.5 (25–72)	22.75 (5–40)	NA	MS: 16.1 (9–27) HC: 24 (10–30)
Vercellino et al. (47)	1. Autoptic series: **Cortex** **Basal** **ganglia** **Thalamus** **Brain stem**	IHC - SYN (confocal microscopy, individual count)	Synaptic vesicle (presynapse)	MBP	Reduction in DM (vs. M)	MS: 9 (7 female; cases have been selected for the presence of active demyelinating gray matter lesions) HC: 11 (3 female)	NA	MS: 47.3 (40–64) HC: 73.5 (35–93)	21.4 (16–36) (including 7 patients, 2 NA)	7.7 (7.0–9.0) (including 5 patients, 4 NA)	MS: 13.8 (7–27) HC: 19.4 (5–33)
	2. Autopic series: **Cortex** - inferior frontal sulcus-superior temporal sulcus	IHC - SYN (confocal microscopy)	Synaptic vesicle (presynapse)	MBP	Reduction in DM (vs. HC)	MS: 7 MS (6 female) HC: 7 (4 female)	NA	MS: 52.3 (39–66) HC: 56.3 (47–65) (including 6 patients, 1 NA)	15.7 (8–29)	7.2 (6.0–9.0) (including 5 patients, 2 NA)	MS: 29.2 (27–30) (including 6 patients, 1 NA) HC: 29.0 (26–34 (including 6 patients, 1 NA)

* *If information was available, the given population represents the subjects in which synaptic measurements were done. Mean values were adjusted as far as possible*.

*DM, demyelinated area; EDSS, expanded disability status scale; HC, healthy control (used also for a person without neurological disability); IHC, immunohistochemistry; M, myelinated area; MBP, myelin basic protein; MOG, myelin oligodendrocyte glycoprotein; MS, multiple sclerosis; NA, no data available; OD, optical density; PLP, myelin proteolipid protein; PPMS, primary progressive multiple sclerosis; RRMS, relapsing remitting multiple sclerosis; RT-PCR, real-time PCR; SPMS, secondary progressive multiple sclerosis; SYN, synaptophysin; vs., versus*.

#### Studies Reporting Synaptic Loss

In the reconstruction of single cortical projection neurons of eight patients with MS and eight healthy controls (HCs), using Golgi-Cox staining and confocal microscopy, Jürgens et al. (17) revealed significant loss of dendritic spines in demyelinated cortex and in NAGM compared to HCs. The spine density/μm was examined in relation to the distance from the cell soma in samples of three different areas of the cortex, namely the insular lobe, the frontotemporal lobe, and the occipital lobe. While in the occipital lobe, a tendency toward spine density reduction in NAGM and lesional GM did not reach significant levels, the relative spine density experienced a drop of at least 50% in both, NAGM and lesional GM in the insular and frontotemporal lobe in the maximal considered distance from the soma of 250 μm (*p* < 0.001). A general trend toward a growing difference of spine density between NAGM and lesional GM to HCs was visible with increasing distance from the soma. Interestingly, significant axonal loss was reported only from lesional GM and not from NAGM. In other words, NAGM experienced a loss of dendritic spines, whereas its density of axons remained unaltered (17).

Wegner et al. (13) reported a reduction of 47% of the mean synaptophysin signal in leucocortical lesions (14.9 ± 8.4 nCi/g tissue equivalent) compared to NAGM (28.16 ± 11.2 nCi/g tissue equivalent; *p* = 0.001) (13). To quantify the synaptic density, immunoautoradiography (using primary anti-synaptophysin antibody and 35S-anti-mouse secondary antibody) was applied to tissue of nine patients with MS and nine HCs. Samples were obtained from the superior frontal gyrus, the precentral, and the postcentral gyrus. The synaptophysin signal appeared not to be significantly different between NAGM and HC. Notably, the synaptic loss (−47%) exceeded the neuronal loss, which was found to be about −10% (leucocortical lesions in relation to normal appearing MS cortex; *p* = 0.032). Furthermore, the mean neuronal cell size in leucocortical lesions was smaller than in NAGM (−9%, *p* = 0.005) (13).

In a recent study, Vercellino et al. (44) observed a decrease of presynaptic input in lesional GM and NAGM. The study was carried out in two autopsy series including all together 16 patients with MS and 18 HCs. Synapses were visualized in applying synaptophysin immunohistochemistry, confocal microscopy, and count of individual synapses. The first autopsy series focused on the comparison of synapse numbers in active GM lesions, inactive chronic GM lesions, and NAGM. Brain tissue was obtained not only from cortical regions but also from the basal ganglia, the thalamus (only tissue from one patient), and the brain stem (only from one patient). Active GM lesions showed a presynaptic loss of 58.9% compared to NAGM (18.5 ± 11.6 synapses/100 μm^2^ in active GM lesion compared to 45.1 ± 12.9 synapses/100 μm^2^ in NAGM; *p* = 0.001). On the other hand, chronic inactive GM lesion presented only a slight presynaptic decrease of 12.6% compared to NAGM (41.8 ± 18.2 synapses/100 μm^2^ in inactive chronic GM lesion vs. 47.8 ± 19.7 synapses/100 μm^2^ in NAGM; *p* = 0.01). Furthermore, the synaptic density in the NAGM of patients with MS was reduced compared to HCs in both, the first and the second autopsy series. The first autopsy series found a decrease of 23.5% of synaptic input in NAGM compared to HCs (mean 46.1 ± 15.5 synapses/100 μm^2^ in NAGM in comparison to 60.3 ± 10.4 synapses/100 μm^2^ in HCs; *p* = 0.001). The second autopsy series examined MS tissue from the layer V of the cerebral cortex taken from the inferior frontal sulcus and the superior temporal sulcus. It yielded comparable results with a reduction of synaptic input in NAGM of 19.5% compared to HCs (mean 54.6 ± 4.1 synapses/100 μm^2^ in NAGM vs. 67.9 ± 9.1 synapses/100 μm^2^ in HCs; *p* < 0.001). Furthermore, the degree of presynaptic loss appeared not to be correlated to neuronal or axonal loss in lesional and NAGM. Notably, this study reported an affection of glutamatergic and gamma-aminobutyric acid (GABA)ergic presynaptic terminals to a similar extend (44).

#### Studies Reporting No Overall Synaptic Loss

In an earlier study, Vercellino et al. (46) examined whole cortical sections of six patients with MS and reported no difference in the optical density (OD) of synaptophysin in demylinated and adjacent NAGM. In a subsequent study of the same research group, the OD of synaptophysin immunoreactivity in cortical lesions of 10 patients with MS (pre-frontal cortex, temporal cortex, insula, and cingulate cortex) was focally decreased compared to NAGM in the presence of a high density of activated microglia (HLA-DR-positive). Demyelinated areas with a comparable density of HLA-DR-positive microglia to NAGM (which accounted for about 95% of the total area of demyelinated cortex in the coronal section) showed no alteration (47).

In a recent study by van Olst et al. (45), the overall synaptic density in the cortical layer 3 of the cingulate and insular cortex was shown to be unaltered, despite strong evidence for almost complete loss of synaptic input in colocation to microglia and presynaptic phagocytosis. Brain tissue was received from 20 patients with secondary-progressive MS and six HCs. Presynapses, marked with synaptophysin, were detected with confocal microscopy.

### Hippocampus

Three studies, comprising 98 patients with MS and 28 HCs, have examined the synaptic density in the hippocampus (Table 2). All of them used synaptophysin as an immunohistochemical marker (11, 12, 18). One of the studies additionally measured the expression of proteins enriched in synaptic terminals (11).

**Table 2:** An overview of publications that investigated the synaptic density in the hippocampus along with extracted data.

**Publication**	**CNS area**	**Synaptic staining**	**Investigated anatomical subregion**	**Myelin detection**	**Result**	Population^*^	**Specifics MS**	**Mean-age (years)**	**Mean disease duration (years)**	**Disability (EDSS)**	**Post-mortem interval (h)**
Papadopoulos et al. (12)	**Hippocampus** - CA1 - CA2/3 - CA4 - subiculum - entorhinal cortex	IHC - SYN (OD)	synaptic vesicle (presynapse)	MBP	reduction in CA4 vs. controls	MS: 45 (32 female) HC: 7	38 SPMS, 7 PPMS	MS: 61 ± 12.9 HC: 63 ± 15.1	27 ± 10.7	NA	MS: 19 ± 10.4 HC: 20.1 ± 7
Dutta et al. (11)	**Hippocampus** - overall - CA1 - DG		pre- and post-synapse, adhesion molecules	PLP		MS: 22 MS overall HC: 9 HC overall	17 SPMS, 5 PPMS; 10 M, 12, DM	MS: 57.7 ± 8.3 HC: 66.3 ± 11.9	26.6 (9–46)	8.5 ± 1.0	MS: 5.9 ± 2.4 HC: 15.5 ± 9.7
		RT-PCR				MS: 10 HC: 5	6 SPMS, 4 PPMS; 5 M, 5 DM				
		Microarray				MS: 8 HC: 4, age-matched	5 SPMS, 3 PPMS; 4 M, 4 DM				
		Western Blot - SYN - synapsin - SV2 - neurexin-1 - neuroligin-1 - PSD-95 - CASK - MAP2			reduction in DM vs. controls	MS:6 HC: 3	5 SPMS, 1 PPMS; 3 M, 3 DM				
		IHC - SYN (confocal microscopy)			reduction in CA 1 and DG (DM vs. M)	MS: 6 HC: 0	3 M, 3 DM				
Michailidou et al. (18)	**Hippocampus** - overall - granular / molecular layer of dentate gyrus - hilus of dentate gyrus - CA1 - CA 3/2 - subiculum	IHC - SYN (confocal microscopy)	synaptic vesicle (presynapse)	PLP	overall reduction (D vs. controls)	MS: 31 overall HC: 12 HC overall, age-matched	20 SPMS and PPMS; 11 M, 8 DM, 1 excluded	MS: 65.2 ± 9.5(M) 63.6 ± 5.4(DM) HC: 68.9 ± 14.3	21.5 (2-59)(including 28 patients where information was available)	NA	MS: 7.1 ± 1.6 (M) 6.9 ± 1.9 (DM) HC: 8.9 ± 4.8
		Western Blot				MS: 11 HC: 4	4 M, 7 DM				

* *If information was available, the given population represents the subjects in which synaptic measurements were done. Mean values were adjusted as far as possible*.

*DM, demyelinated area; EDSS, expanded disability status scale; HC, healthy control (used also for person without neurological disability); IHC, immunohistochemistry; M, myelinated area; MBP, myelin basic protein; MOG, myelin oligodendrocyte glycoprotein; MS, multiple sclerosis; NA, no data available; OD, optical density; PLP, myelin proteolipid protein; PPMS, primary progressive multiple sclerosis; RRMS, relapsing remitting multiple sclerosis; RT-PCR, real-time PCR; SPMS, secondary progressive multiple sclerosis; SYN, synaptophysin; vs., versus*.

#### Hippocampus Overall

Out of three studies that examined synaptophysin in the hippocampus, one study could show an overall four-fold decrease of synaptophysin immunoreactivity in lesional MS hippocampi (*p* < 0.0001) compared to HCs (18). Significant results were furthermore reported from hippocampal subfields. One study performed protein analysis with samples from three myelinated and three demyelinated MS hippocampi and three HCs (11). Specifically, mRNA levels and associated synaptic proteins of synaptophysin, synaptotagmin, SV2, the adhesion molecules neurexin-1 and neuroligin-1, the PSD-95, the calcium/calmodulin-dependent serine protein kinase (CASK), and some housekeeping genes, including MAP2, were taken into account (11). The results yielded a significant intensity reduction for synaptic proteins in demyelinated hippocampi compared to HCs (HC intensity is set to be =1) (Table 3). Synaptophysin and synapsin reached 0.88 and 0.74, respectively, of the intensity of the HCs in demyelinated hippocampi. Respective protein levels of myelinated hippocampi showed no significant alterations (*p* < 0.05). The adhesion molecules neurexin-1 and neuroligin-1 were reduced to 0.35 (*p* < 0.05) and 0.51 (*p* < 0.00005) in demyelinated areas compared to HCs. On the other hand, neuroligin showed an increase to 2.06 in the myelinated hippocampi compared to HCs (*p* < 0.00005). PSD-95 and CASK experienced a decrease in the demyelinated hippocampi compared to HCs to 0.21 and 0.62, respectively (*p* < 0.005). Furthermore, for CASK, a reduction to 0.80 was apparent also in myelinated hippocampi compared to HCs (*p* < 0.005). In neurexin-1 and PSD-95, protein levels in myelinated hippocampi appeared not to be significantly altered. SV2 levels showed no significant changes in MS hippocampi compared to HCs, and a tendency toward an increase of MAP2 levels in demyelinated MS hippocampi compared to myelinated MS hippocampi did not reach significance (11).

**Table 3:** The intensity of different proteins measured in the hippocampus by Dutta et al. (11).

**Synaptic protein**	**HC intensity**	**MS-M intensity compared to HC**	**MS-DM intensity compared to HC**
SYN	1	1	0.881
SYT	1	0.977	0.739
SV 2	1	1.042	0.953
Neurexin-1	1	1.056	0.35
Neuroligin-1	1	2.051	0.51
PSD-95	1	0.804	0.21
CASK	1	0.797	0.622

*Red filled cells represent significantly altered results. Gray cells represent no significant alteration*.

*CASK, calcium/calmodulin-dependent serine protein kinase; HC, healthy control (used also for person without neurological disability); MS-M, myelinated areas of patients with MS; MS-MD, demyelinated areas of patients with MS; PSD-95, postsynaptic density protein 95; SV2, synaptic vesicle protein 2; SYN, synaptophysin; SYT, synaptotagmin*.

#### CA 1 and Subiculum

Immunohistochemical analysis of synaptophysin showed a significant decrease in the demyelinated CA1 subfield in two studies (11, 18) and a non-significant tendency toward reduction in a third study (12). Immunoreactivity for synaptophysin was assessed percentage-wise of immunoreactive area over the total area. Dutta et al. (11) reported a 1.85-fold (*p* = 10^−9^) drop of synaptophysin positive punctae in the demyelinated CA1 subfield, notably compared to myelinated MS hippocampi. Michailidou et al. (18) found a 4.5-fold (*p* < 0.01) reduction of synaptophysin immunoreactivity in the demyelinated CA1 and 4.9-fold decrease (*p* < 0.05) in the subiculum compared to HCs. One study suggested a tendency toward reduction in CA1 and subiculum also in myelinated hippocampi; however, statistical significance was not reached.

#### CA 3/2 and CA 4 (Hilus of the Dentate Gyrus)

Two studies specified measurements of synaptophysin in the CA 2/3 and CA 4 subfields. While on the one hand, a significant 4.4-fold decrease (*p* < 0.05) of synaptophysin immunoreactivity in demylinated CA 2/3 MS hippocampi compared to HCs was reported (18), a tendency toward decrease of the OD of synaptophysin in MS hippocampi did not deviate significantly in another study (12). In CA 4, the OD of synaptophysin in MS hippocampi was shown to be significantly reduced by 46.1% (*p* = 0.014) compared to HCs. Furthermore, the loss of synaptic terminals appeared to be uncorrelated to demyelination and neuronal loss (12). The results are supported by the tendency toward loss of synaptic terminals in myelinated and demyelinated areas compared to HCs in another study; however, the results failed to be statistically significant (18).

#### Dentate Gyrus

A 2.4-fold (*p* = 0.0049) drop of synaptophysin immunoreactivity in the demyelinated dentate gyrus of MS hippocampi, notably compared to myelinated hippocampi (11) and a non-significant tendency toward synaptic loss in myelinated and demyelinated MS hippocampi compared to HCs was reported (18).

### Cerebellum

Three studies, comprising 50 patients with MS and 19 HCs, have analyzed the synaptic density in the cerebellum (20, 48, 49), one of which found a significant change (20) (Table 4). One study, including 16 patients with MS and eight HCs, focused specifically on synapses in the dentate nucleus and reported synaptic loss, irrespectively of focal demyelination (20). The number of synapses was determined by counting synaptophysin-positive punctae at the soma membrane of neurons using light microscopy and confocal microscopy. Furthermore, the synaptic density as synapses/μm^2^ was explored. Overall, the number of synaptic structures per neuron, and the mean synaptic density appeared to be significantly lower in patients with MS compared to HCs, irrespectively of focal demyelination (20). Moreover, the number of neurons/mm was significantly reduced in demyelinated, but not in myelinated areas of patients with MS compared to HCs (20).

**Table 4:** An overview of publications that investigated the synaptic density in the cerebellum, the thalamus, and in the spinal cord, along with extracted data.

**Publication**	**CNS Area**	**Synaptic Staining**	**Investigated anatomical subregion**	**Myelin Detection**	**Result**	Population^*^	**Specifics MS**	**Mean-age (years)**	**Mean Disease Duration (years)**	**Disability (EDSS)**	**Post-mortem interval (h)**
Kutzelnigg et al. (48)	**Cerebellum** - granule cell layer	IHC - SYN (OD)	Synaptic vesicle (presynapse)	PLP	No change	MS:7 HC: 0	7 M	NA	NA	NA	NA
Howell et al. (49)	**Cerebellum** - granule cell layer - molecular layer	IHC - SYN (OD)	Synaptic vesicle (presynapse)	MOG	No change	MS: 27 (19 female) HC: 11 (8 female)	27 SPMS	MS: 53.4 (30–64) HC: 66.5 (35–84)	24.1 (6–40)	NA	MS: 25.7 ± 4 HC: 21.5 ± 4.1
Albert et al. (20)	**Cerebellum** - dentate nucleus	IHC - SYN (confocal microscopy, light microscopy, manual count)	Synaptic vesicle (presynapse)	MBP, PLP	Reduction in M and DM	MS: 16 MS overall (6 female) HC: 8 HC overall (gender NA), age-matched	2 Acute MS, 1 RRMS, 5 SPMS, 6 PPMS, 2 NA; 9 M, 7 DM	MS: 53 (36–69) (including 15 p., 1 NA) HC: NA	11.9 (<1–30) (including 12 patients, 4 NA)	NA	NA
		Electron microscopy			Suggests lysosomal degradation of synapses	MS: 1 (female) HC: 1 (male)	1 SPMS	MS: 39 HC: 55	14	NA	NA
Wernburg et al. (19)	**Thalamus** - lateral geniculate nucleus (retinogeniculate system)	IHC (using confocal microscopy) - VGluT2	Presynapse		Reduction compared to controls	MS: 5 (3 female) HC: 5 (2 female)	4 progressive MS, 1 PPMS	MS: 70.4 (59–78) HC: 67.4 (58–84)	NA	NA	MS: 12.8 (7–24) HC: 32.6 (24–50)
Petrova et al. (21)	**Spinal cord** - cervical, thoracic and lumbar cord levels	IHC - SYN - Synapsin 1	Synaptic vesicle (presynapse)	MBP	Reduction in M and DM	MS: 15 HC: 7	NA	MS: 69 ± 11 HC: 82 ± 7	29 ± 11	NA	MS: 36 ± 17 HC: 36 ± 17

* *If information was available, the given population represents the subjects in which synaptic measurements were done. Mean values were adjusted as far as possible*.

*DM, demyelinated area; EDSS, expanded disability status scale; HC, healthy control (used also for person without neurological disability); IHC, immunohistochemistry; M, myelinated area; MBP, myelin basic protein; MOG, myelin oligodendrocyte glycoprotein; MS, multiple sclerosis; NA, no data available; OD, optical density; PLP, myelin proteolipid protein; PPMS, primary progressive Multiple Sclerosis; RRMS, relapsing remitting multiple sclerosis; RT-PCR, real-time PCR; SPMS, secondary progressive multiple sclerosis; SYN, synaptophysin; vs., versus*.

In another study, no significant difference was reported from the comparison of the density of anti-synaptophysin immunoreactivity between myelinated and demyelinated areas of the granule cell layer of the cerebellum in the tissue of seven patients with MS (48). Similarly, no significant alterations appeared in the comparison of the intensity of anti-synaptophysin immunoreactivity in the granule cell layer and the molecular layer to HCs (49).

### Thalamus

Werneburg et al. (19) examined quantitative changes of presynaptic input in the human lateral geniculate nucleus of the thalamus (Table 4). The lateral geniculate nucleus receives synaptic input from retinal ganglion cells, which can be specifically visualized using VGluT2 immunoreactivity. Five patients with progressive MS and five HCs were included in the analysis with anti- VGluT2 immunostaining and confocal microscopy. A two-fold reduction of immunostained area (3.9% in MS vs. 1.9% in HCs) was detected in patients with MS compared to HCs (*p* < 0.0001). No alteration was found for the PSD-95 protein (19).

### Spinal Cord

One study, comprising 15 patients with MS and seven HCs, examined the synaptic density in the spinal cord (21) (Table 3). Immunohistochemical measurements for synaptophysin and synapsin were performed in lesional and NAGM. The visual staining intensity for synaptophysin appeared to be reduced in lesional (58%, *p* < 0.001) and in NAGM (54%, *p* < 0.001) compared to HCs. A significant difference was also seen between lesional and NAGM (9%, *p* = 0.004). Furthermore, Petrova et al. (21) explored the synaptic bouton area (μm^2^) on a mean of five neurons (immunostaining for synaptophysin) and reported a remarkable reduction within GM lesions (96%, *p* < 0.001) and in NAGM (92%, *p* < 0.001) compared to HCs. The comparison of lesional GM to NAGM yielded a significant decrease of 42% (*p* < 0.001). Similar results were found with immunostaining for synapsin-1 (reduction of synaptic bouton area of 58% compared to HCs, *p* < 0.001). While in HCs, the synaptic bouton and anterior horn GM area and the number of neurons were correlated (*p* < 0.0001), in MS spinal cords a correlation was not visible. Similarly, while the number of neurons and the synaptic bouton area showed a correlation in HCs (*p* < 0.001), no correlation was found in patients with MS.

## Discussion

This systematic review is a survey of the research literature on synaptic density in the human CNS of patients with MS. Our investigation reveals synaptic loss in various areas of the CNS. However, applied methods and results are inconsistent, and most studies are limited on the visualization of presynaptic structures. Conflicting results in synaptic quantification could be a result of varying measurement techniques to detect not only foremost synaptophysin-immunoreactivity but also other synaptic markers. The most abundant methodological approaches to visualize synaptophysin-immunoreactivity were OD measurements and the use of confocal microscopy. Although OD measurements can reach a higher regional resolution compared to other methods, such as western blot, they appear to have considerable limitations in accurate and sensitive quantification of synapses (27). Indeed, the majority of studies using OD to detect synaptic loss in MS report no significant change (46–49). In contrast, confocal microscopy provides a higher spatial precision compared to light microscopy analysis (50), which could be a reason for higher sensitivity in detecting synaptic loss in studies relying on this technique (17–19). Nevertheless, comparable studies also provided evidence for an unaltered overall synaptic density in areas of the cortex and suggested only focal synaptic disappearance in colocation to activated microglia (45). On the other hand, stereological analysis, where the total number of synapses is estimated based on a subgroup of individually counted stained points, has been demonstrated to be both, highly sensitive and accurate in detecting local differences (27). This might account for the observation of pre- and postsynaptic loss in the groundbreaking studies (17, 20, 44). Furthermore, the included studies have been inconsistent in the choice of a reference region. Some studies have compared measurements in demyelinated GM area solely to NAGM in patients with MS (46, 47), whereas other studies compared lesional and NAGM to HCs. In other words, some studies assumed an unaltered histological environment in GM areas without signs of demyelination. Since we know that considerable pathology takes place also in the NAGM (17, 44), this might have led to false conclusions, most likely to an overestimation of the synapse number in demyelinated areas. Importantly, the majority of studies measured solely presynaptic protein levels. Few human studies have taken other anatomical substructures, such as the synaptic gap and the postsynaptic site, into account (11, 17). Additional research is required in order to draw reliable conclusions about the correlation between loss of presynaptic proteins and quantitative changes of whole synaptic connections in MS. Partly, more precision is also needed in the specification of the targeted molecule. For example, Dutta et al. (11) reported no alteration in the density of SV2, a synaptic vesicle protein in the presynapse. However, SV2 comprises the three isoforms SV2A, SV2B and SV2C, out of which only SV2A is ubiquitously expressed throughout the adult brain. The expression of SV2B and SV2C is much more variable (30), a fact which needs to be taken into account for drawing valid conclusions.

Mechanisms leading to synaptic loss are likely multifactorial. Several mechanisms have been proposed; nevertheless, a comprehensive understanding is still missing. Consistent with our findings, demyelination has a notable impact on the synapse number. Demyelination leads to an altered gene expression; it is disrupting the physiological microenvironment and contributes to a decrease of essential synaptic proteins (11). Closely related is the excitotoxic hypothesis, which states that an environment of enhanced glutamate and reduced GABA is having a negative impact on synapses. This is supported by the observation that the selective inhibition of AMPA receptors in experimental autoimmune encephalomyelitis (an animal model for MS) can prevent clinical disability and dendritic spine loss (51, 52). Further involved processes might be neuron-autonomous synaptic elimination (20) and trans-synaptic anterograde and retrograde synaptic degeneration as a consequence of lesional areas (20, 53). Additionally, histopathological studies have pointed toward a complement-assisted (C1q-C3) elimination of synapses by microglia in a mildly inflamed environment (18). This process is of special interest because it has been discovered also in NAGM (18). Reports of lesion-independent synaptic loss, which can surpass axonal loss, give rise to the question of a primary process leading to a reduction of synapses (17). Complement-assisted elimination of synapses by microglia appears to have similarities to the physiological pruning of synapses throughout life, an essential mechanism to ensure functional neuronal circuits (54, 55).

The quantification of synapses in post-mortem studies has clearly its limitations. Subjects are for the most part in advanced stages of the disease, and dynamic processes are difficult to unravel. Animal models used to study MS, such as the experimental autoimmune encephalomyelitis (EAE), are therefore an important tool. Indeed, EAE studies have demonstrated alterations in the synaptic density to be a dynamic process. Synapse numbers are suggested to partly normalize completely after the recovery from EAE (56). Evidence for regeneration is also visible in human MS studies, such as an increased ratio of GAP-43/SYN in leucocortical lesions (GAP-43 is expressed in growth clones and axons). A relative increase of GAP-43 might indicate local remodeling processes (13). Also when compared to traumatic injury, leucocortical lesions of early MS appear to present a higher regeneration potential (57). To which extent synaptogenesis is part of a regeneration process in MS is still elusive. However, mechanisms involved in synaptic elimination and regeneration might be a suitable target for therapeutic interventions. Therefore, new methods to study the synaptic density directly in humans in the living organism are urgently needed. A promising approach is a recently introduced PET imaging technique using the synaptic vesicle glycoprotein 2A (SV2A)-binding radioligand ^11^C-UCB-J, which can be used to visualize the synaptic density *in vivo* (58). With this approach, changes in synaptic density have been reported from several neurological and psychiatric diseases, such as Parkinson's, dementia with Lewy bodies (59, 60), Alzheimer's disease (61–63), temporal lobe epilepsy (58, 64), major depressive disorder (65), post-traumatic stress disorder (65), and schizophrenia (66). Comparable *in vivo* studies still need to be carried out for MS.

## Conclusion

We have found evidence for widespread synaptic loss throughout the MS brain and in the MS spinal cord, partly exceeding lesional areas. However, results are inconsistent, probably due to differences in the applied methods. We have found a tendency toward an increased methodological effort to tackle the question of synaptic loss in MS. In order to gain a better understanding of the underlying pathomechanisms and dynamic processes, further research and *in vivo* studies are needed.

## Data Availability Statement

The original contributions presented in the study are included in the article/supplementary material, further inquiries can be directed to the corresponding author/s.

## Author Contributions

EM did the study design, data extraction, drafting, and revision of the manuscript, as well as the screening and selection of potential publications. EH contributed to the screening and selection of potential publications. LA carried out the study supervision and participated in the revision of the manuscript. All authors contributed to the article and approved the submitted version.

## Funding

This work was supported by Sigrid Juselius Foundation.

## Conflict of Interest

The authors declare that the research was conducted in the absence of any commercial or financial relationships that could be construed as a potential conflict of interest.

## Publisher's Note

All claims expressed in this article are solely those of the authors and do not necessarily represent those of their affiliated organizations, or those of the publisher, the editors and the reviewers. Any product that may be evaluated in this article, or claim that may be made by its manufacturer, is not guaranteed or endorsed by the publisher.

## Abbreviations

CNS: central nervous system
EAE: experimental autoimmune encephalomyelitis
GM: gray matter
HC: healthy control (not suffering from any other neurological condition)
MS: multiple sclerosis
NAGM: normal appearing gray matter
SV2A: synaptic vesicle glycoprotein 2A.
